# Fabrication and Analysis of Near-Field Electrospun PVDF Fibers with Sol-Gel Coating for Lithium-Ion Battery Separator

**DOI:** 10.3390/membranes11030186

**Published:** 2021-03-09

**Authors:** Mark D. Francisco, Cheng-Tang Pan, Bo-Hao Liao, Mao-Sung Wu, Ru-Yuan Yang, Jay CJ Chu, Zhi-Hong Wen, Chien-Feng Liao, Yow-Ling Shiue

**Affiliations:** 1 Institute of Biomedical Sciences, NSYSU, Kaohsiung City 80424, Taiwan; markfrancisco_trinity@mem.nsysu.edu.tw; 2 Department of Mechanical and Electro-Mechanical Engineering, National Sun Yat-sen University (NSYSU), No. 70, Lienhai Rd, Gushan District, Kaohsiung City 80424, Taiwan; pan@mem.nsysu.edu.tw (C.-T.P.); liaobo0411@gmail.com (B.-H.L.); 3 Department of Chemical and Materials Engineering, National Kaohsiung University of Science and Technology, No. 415, Jiangong Rd., Sanmin District, Kaohsiung City 80778, Taiwan; mswu@nkust.edu.tw; 4 Department of Science and Technology, National Pingtung University, No.4-18, Minsheng Rd., Pingtung City 900391, Taiwan; ryyang@mail.npust.edu.tw; 5 Green Epoxy Technology, Inc., Anaheim, CA 92802, USA; jaycjchu@gmail.com; 6 Department of Marine Biotechnology and Resources, National Sun Yat-sen University, No. 70, Lienhai Rd, Gushan District, Kaohsiung City 80424, Taiwan; wzh@mail.nsysu.edu.tw; 7 Department of Emergency Medicine, Kaohsiung Armed Forces General Hospital, Kaohsiung City 80284, Taiwan; 8 Institute of Precision Medicine, NSYSU, Kaohsiung City 80424, Taiwan

**Keywords:** near-field electrospinning, PVDF membrane, lithium-ion battery, separator, sol-gel

## Abstract

Environmental and economic concerns are driving the demand for electric vehicles. However, their development for mass transportation hinges largely on improvements in the separators in lithium-ion batteries (LIBs), the preferred energy source. In this study, innovative separators for LIBs were fabricated by near-field electrospinning (NFES) and the sol-gel method. Using NFES, poly (vinylidene fluoride) (PVDF) fibers were fabricated. Then, PVDF membranes with pores of 220 nm and 450 nm were sandwiched between a monolayer and bilayer of the electrospun fibers. Nanoceramic material with organic resin, formed by the sol-gel method, was coated onto A4 paper, rice paper, nonwoven fabric, and carbon synthetic fabric. Properties of these separators were compared with those of a commercial polypropylene (PP) separator using a scanning electron microscope (SEM), microtensile testing, differential scanning calorimetry (DSC), ion-conductivity measurement, cyclic voltammetry (CV), and charge-discharge cycling. The results indicate that the 220 nm PVDF membrane sandwiched between a bilayer of electrospun fibers had excellent ionic conductivity (~0.57 mS/cm), a porosity of ~70%, an endothermic peak of ~175 °C, better specific capacitance (~356 mAh/g), a higher melting temperature (~160 °C), and a stable cycle performance. The sol-gel coated nonwoven fabric had ionic conductivity, porosity, and specific capacitance of ~0.96 mS/cm., ~64%, and ~220 mAh/g, respectively, and excellent thermal stability despite having a lower specific capacitance (65% of PP separator) and no peak below 270 °C. The present study provides a significant step toward the innovation of materials and processes for fabricating LIB separators.

## 1. Introduction

Owing to environmental issues and concerns as well as high global energy prices, the value of driving electric vehicles has been recognized, resulting in gradual market expansion [1]. However, the biggest bottleneck in the further development of electric vehicles is the fabrication and production of batteries with high power density [2]. Currently, prominent car factories have invested substantially in the development and improvement of the power density of batteries for electric vehicles. Of the many batteries that could be used for electric vehicles, lithium-ion batteries (LIBs) offer considerable energy density advantages [3]. In LIBs a separator between the cathode and the anode permits the stable transmission of lithium ions; however, the structure and properties of the separator can affect the safety, cycle life, and energy density of LIBs [4]. Thus, the separator is an important component in innovations to improve the performance and safety aspects of LIBs. At present, considerable efforts are being devoted to improving lithium-ion battery technology, given that its cost is a concern. Consequently, innovations in separator technology can significantly improve the performance of LIBs in electric vehicles.

Commercial separators used in LIBs are typically microporous polyolefin polymer films (polyethylene (PE) and polypropylene (PP)) because of their high mechanical strength, good electrochemical and chemical stability, low cost, and thermal shutdown property [5,6,7]. Additionally, they provide films of uniform pore size [8]. Nevertheless, they present some disadvantages when used in state-of-the-art energy storage devices and electric or hybrid-electric vehicles [9]. When the temperature of an LIB rises to the melting point of the separator, the latter starts to melt [10]. Moreover, a separator’s intrinsic hydrophobicity and low surface energy result in poor wettability for the polar liquid electrolyte, thus restricting its ability to transmit power LIBs [11]. Nanoparticle inorganic metal oxides are combined with Sol-gel technology to make inorganic composite membranes, which are known to have high thermal stability and conductivity, characteristics that can improve the safety of the battery. In addition, the puncture resistance of a membrane is better than that of a polymer film but not as tough.

Although non-polar polyolefin separators are inexpensive, they can shrink easily in response to heat, which raising safety concerns when using the battery. In addition, their electrolyte absorption is low.

Nonwoven fabric membranes have been developed for LIB separators, but they are still not widely used because of the difficulty producing thin membranes with good uniformity and high strength. Therefore, they are usually coated with ceramic material to improve thermal strength and stability. In contrast, electrospun nonwoven electrolytic membranes have high ionic conductivity, good electrochemical properties, and excellent porosity due to their unique porous structure [12].

Poly (vinylidene fluoride) (PVDF) is a high molecular-weight polymer. Its copolymer has a high polarity and dielectric constant compared to that of PP and PE [13]. Controllable fibers with diameters ranging from a few micrometers to even a few nanometers can be fabricated using near-field electrospinning (NFES) [14,15]. But unlike conventional electrospinning, NFES needs only a short distance to produce continuous fibers.

This study sought to fabricate separators for lithium-ion battery with comparable or better properties than those of commercial PP separator. PVDF membranes with pore sizes of 220 nm and 450 nm were sandwiched in a monolayer and bilayer of PVDF fibers fabricated by near-field electrospinning (NFES). In addition, nanoceramic material, prepared using the sol-gel method was coated onto A4 paper, rice paper, nonwoven fabric, and carbon synthetic fabric. The structural, electrochemical and thermal properties of these separators were investigated and compared with those of commercially available PP separators.

## 2. Materials and Methods

Poly (vinylidene fluoride) (PVDF) fibers were fabricated by near-field electrospinning (NFES) process and used to make a monolayer and bilayer to sandwich PVDF membranes with pore size of 220 nm and 450 nm. However, the sol-gel was coated on the surfaces of four substrates: A4 paper, rice paper, nonwoven fabric, and carbon synthetic fabric. Their structural and electrochemical properties were analyzed and compared with those of the commercially available PP separators. The PVDF material was purchased from Sigma-Aldrich Lot number 182702, St. Louis, MO USA, with average Mw ~534,000 in powder format.

### 2.1. Solution Configuration

PVDF powder was blended with acetone in a bottle and labelled as Solution A. The powder was dispersed uniformly in acetone. Dimethyl sulfoxide (DMSO) was blended with a surfactant in a bottle and designated as Solution B. Finally, Solution A was blended with Solution B and stirred for 60 min at 500 rpm. The chemicals used to make the two solutions are presented in Table 1. The ratios of the components of the PVDF solutions are presented in Table 2.

### 2.2. Fabrication of PVDF Fibers by Near-Field Electrospinning (NFES) Process

**Figure 1:**
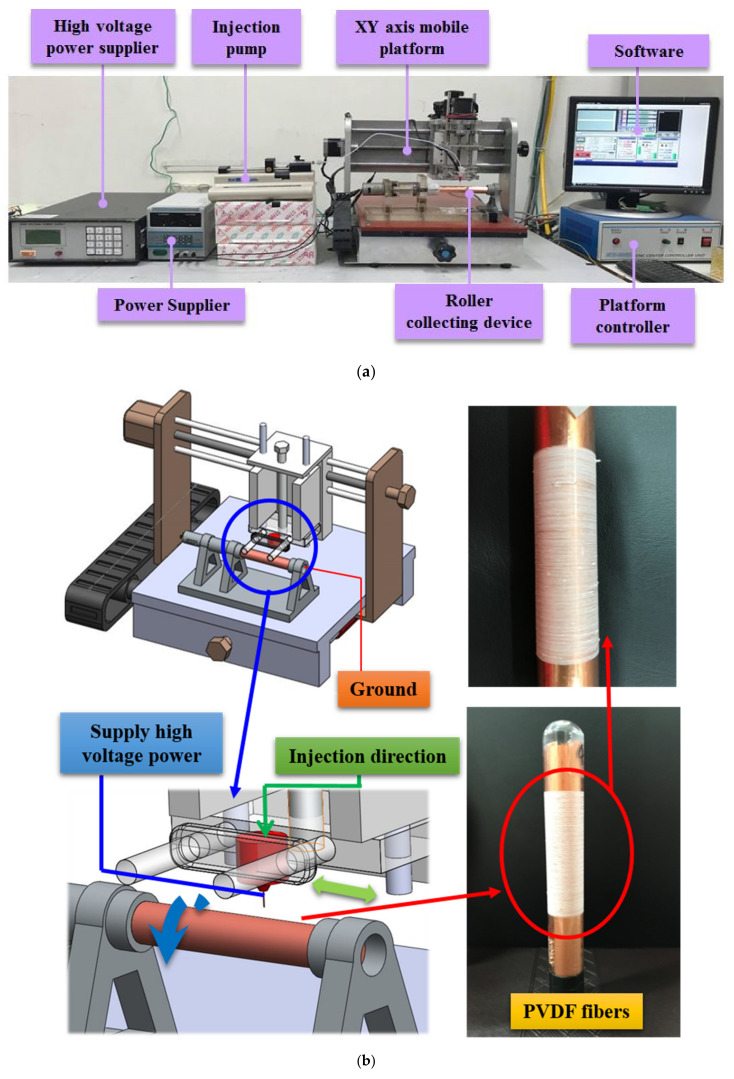
Near-field electrospinning (NFES) equipment. (**a**) experimental set up (**b**) schematic experimental illustration.

As shown in Figure 1, the NFES equipment/set-up includes a high-voltage power supply, XY axis mobile platform, injection pump, roller collecting device, platform controller, and computer software (YOKOGAWA Inc., Tokyo, Japan). In the NFES process, the roller serves as a device for the collection of the PVDF fibers. A copper tape is attached to the glass tube while the grounding end forms a loop on the moving platform. When the motor starts to rotate the glass tube, the collection of PVDF fibers occurs by the movement of the XY axis mobile platform. When high voltage is supplied to the needle, an electric field forms between the solution and the collecting device. The droplets exert an electrostatic force on the needle’s ground port due to the accumulation of charge. As a result, the electrostatic force overcomes the surface tension of the droplets. As the droplets form a Taylor cone, they spray the fibers on the collecting device. The NFES parameters for the fabrication of the PVDF fibers are given in Table 3.

### 2.3. Preparation of PVDF Fiber-Membrane Separators by Sandwich Method

**Figure 2:**
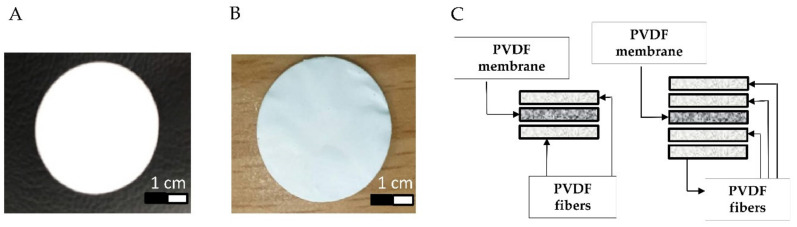
Fabrication of PVDF fiber-membrane separators; (**A**) membrane with pore size of 220 nm, (**B**) membrane with pore size of 450 nm, and (**C**) sandwich technique for preparing monolayer and bilayer fiber-membrane separators.

Two PVDF membranes of varying pore size, namely, 220 nm and 450 nm (see Figure 2A,B), were sandwiched between a monolayer and a bilayer of the fabricated PVDF fibers, as shown in Figure 2C. When the fibers were still in a semi-crystalline state, they were laid above and below the membranes. The resulting separators have a diameter of 18 mm and a thickness of about 120–164 μm. Although the huge thickness of the PVDF separator could prohibit its application to batteries, this study mainly focused on exploring the effectiveness of PVDF film as the coin cell separator. How to reduce the thickness of the PVDF separator is a critical issue for coin cell application. The optimization of the thickness of the PVDF separator will be explored and characterized.

### 2.4. Sol-Gel Preparation and Coating on Substrates: A4 Paper, Rice Paper, Nonwoven Fabric, and Carbon Synthetic Fabric

The B116 solution, consisting of ceramic material composed of alumina (Al_2_O_3_) and silica (SiO_2_), was prepared by the sol-gel method. It was blended with an organic polymer resin at varying ratios (1:1, 3:1 and 9:1) and designated as BX100, BX300, and BX900, respectively. The three solutions were coated separately on the surfaces of the four substrates (A4 paper, rice paper, nonwoven fabric, and carbon synthetic fabric) using a coating bar and subsequently oven-dried. The resulting separators have a diameter of 18 mm.

### 2.5. Porosity Measurement

The porosity measurement was undertaken using the n-butanol impregnation method [16]. The membrane was first immersed in n-butanol for one hour. Subsequently, porosity was calculated using the porosity equation shown in Equation (1), where m_a_ is the mass of n-butanol; ρ_a_ is the density of n-butanol; m_b_ is the mass of the membrane; and ρ_b_ is the density of the membrane.

(1) $$\mathrm{Porosity}\;\left(\%\right)=\frac{\frac{m_{a}}{\rho_{a}}}{\frac{m_{a}}{\rho_{a}}+\frac{m_{b}}{\rho_{b}}}\times 100\%$$

### 2.6. Thermal Stability Determination

Three (3) mg of membrane were placed on an aluminum pan. Then, the aluminum pan was packed by machine and placed into the TA instruments Q20 (Differential scanning calorimetry, DSC). At a nitrogen flow rate of 50 mL/min, the temperature was set for a two-stage temperature rise. The temperature was raised from 40 to 180 °C in a boiler and maintained for 10 min. The results of the analysis were used to determine the crystallinity relationship [17] using Equation (2), where $∆H_{f}$ is the melting enthalpy, and $∆H_{f}^{0}$ is the melting enthalpy of the crystalline polymer. According to the melting temperature, we heated the PP separator and the PVDF fiber-membranes. Subsequently, the PP separator and PVDF fiber-membranes were observed by a scanning electron microscope (SEM).

(2) $$\mathrm{Crystallinity}\;\left(\%\right)=\frac{∆H_{f}}{∆H_{f}^{0}}\;\times 100\%\;$$

### 2.7. Ion Conductivity Measurement

**Figure 3:**
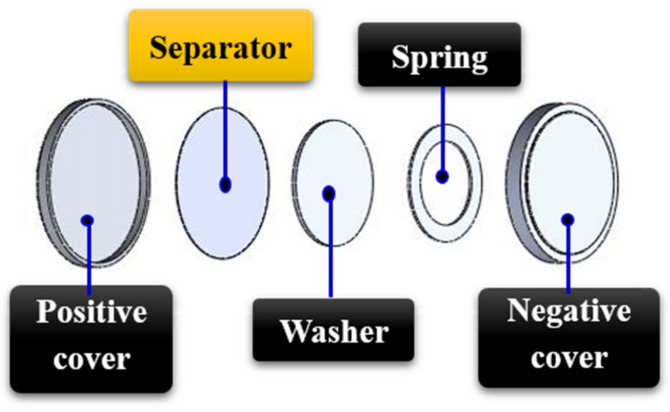
Assembly process of the button battery for ion conductivity determination.

The coin cell consisting of a separator soaked in electrolyte (LiPF6–based solution lot number AD1710, purchased from Eternal Materials Co., Ltd., Hsinchu City, Taiwan) and two symmetrical stainless-steel plates was assembled, as shown in Figure 3. The ionic conductivity of the electrolyte soaked in separator was evaluated using the electrochemical impedance spectroscopy (EIS) measurement (AutoLab, PGSTAT302N, Metrohm) under room temperature. The amplitude was set at 10 mV while the electrical frequency was set at 1 Hz to 1 MHz by applying AC voltage. Finally, the real impedance was calculated using the ion conductivity equation shown in Equation (3), where L is the membrane thickness, R is the resistance, and A is the membrane area.

(3) $$\mathrm{Ion}\;\mathrm{conductivity}\;\left({\mathrm{mS}\;\mathrm{cm}}^{-1}\right)=\frac{L\;\left(\mathrm{cm}\right)\times 1000}{R\;\left(\Omega \right)\times A\;\left({\mathrm{cm}}^{2}\right)}$$

### 2.8. Electrochemical Stability Measurement

**Figure 4:**
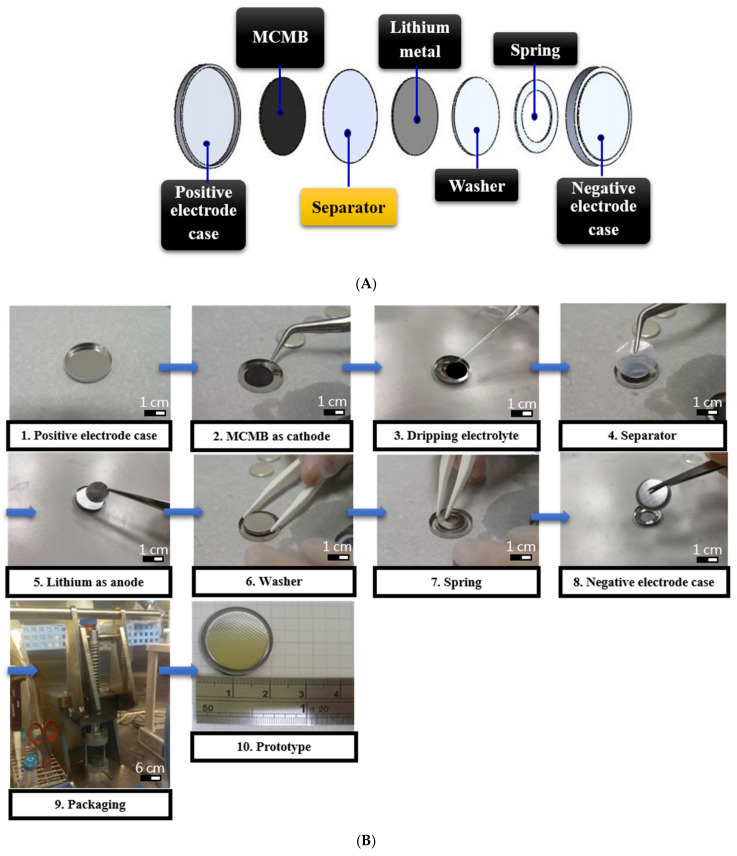
(**A**) Schematic figure, (**B**) assembly process of the coin battery for thermal stability determination.

The coin battery, consisting of a stainless-steel plate, lithium metal, Mesocarbon microbeads (MCMB), electrolyte, and separator, was assembled as shown in Figure 4A. In this study, the MCMB electrode sheets were used as the cathode and lithium metal as the anode. As shown in Figure 4B, there are 10 steps to completing a prototype. First, the positive electrode case was prepared (step 1). Then MCMB as the cathode was attached atop (2), followed by the dripping electrolyte (3). Then the separator was put on the top of the MCMB (4). After that, lithium as the anode was put on top of the separator (5), followed by a washer (6) and spring (7), respectively. Finally, a negative electrode case (8) was put in place to complete the packaging process, (9) all in the glove box. The prototype is shown in (10). When dripping the electrolyte, be aware that too much or too little can affect the yield of the assembled battery. To avoid elemental contact that could cause the battery to short circuit, all parts must be aligned in the center during assembly. A cyclic voltammetry (CV) test was conducted to examine the electrochemical characteristics using CHI 727C purchased from CH Instruments.

The electrochemical stability of the separators was measured by CV. After the battery was placed on the holder, the potential range was scanned from 0.01 V to 3.00 V. CV was performed for three cycles, starting from the open-circuit potential (about 3 V).

### 2.9. Cycle Performance Measurement

The battery-testing system was used to measure battery capacity and cycle life. MCMB served as the working electrode and lithium metal as both the counter and reference electrodes. After placing the battery on the holder, the voltage range was scanned from 0.01 V to 3.00 V. The discharge current density was set at 0.2 C and measured after 21 cycles (1 C = 350 mA/g). The batteries were then cycled at a fixed charge/discharge current density of 0.2 C/0.2 C for a 100-cycle performance testing. The results were subsequently compared with that of the PP separators. MCMB used in the study is with density of ~1.3–1.8 g/cm^3^, thickness of ~30–35 μm, capacity of ≥350 mAh/g, and particle size of ~20–30 μm. It has the advantages of high purity, high crystallinity, low cost, and simple synthesis.

## 3. Results

### 3.1. Surface Analysis of PP Separator and PVDF Fiber-Membranes

**Figure 5:**
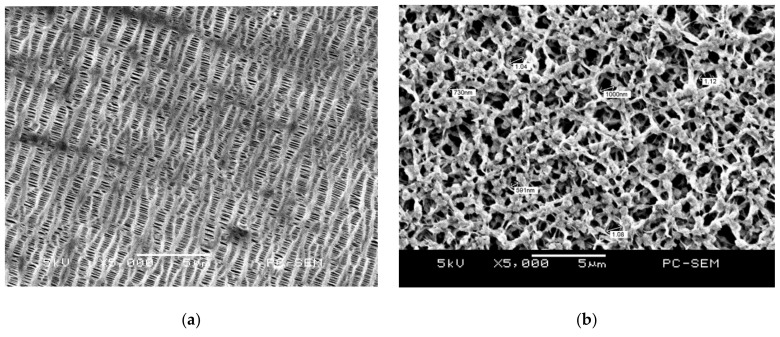

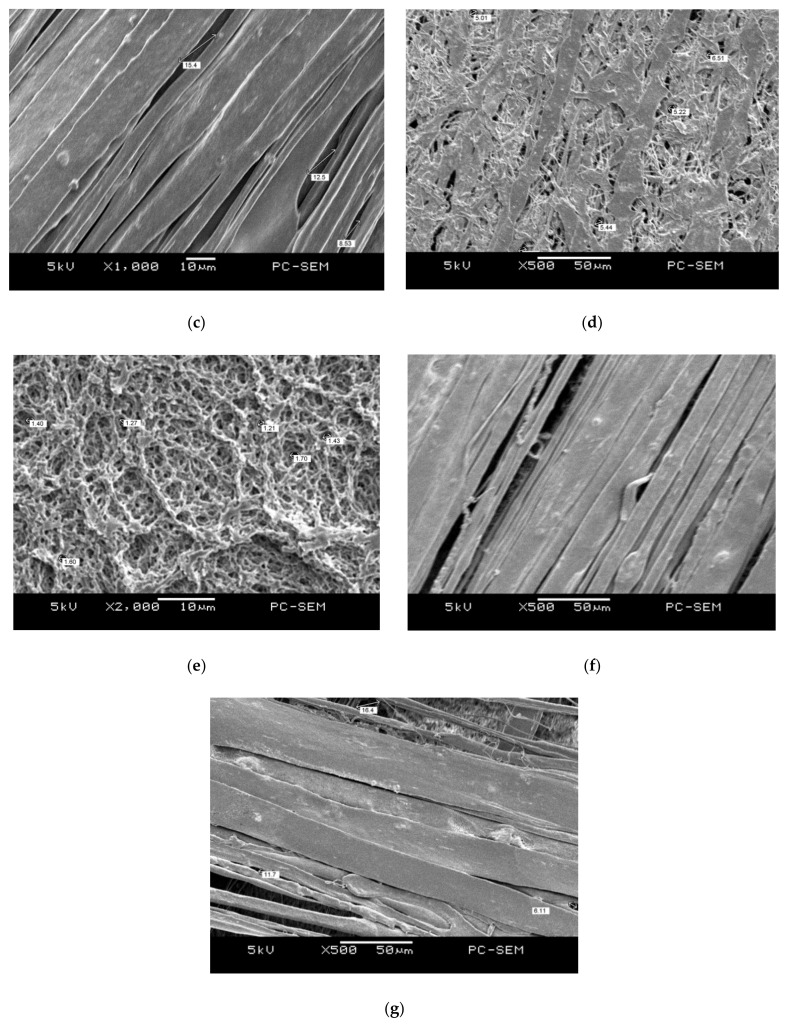
SEM images of PP separators and PVDF fiber-membranes; (**a**) PP separators at 5000× magnification, (**b**) PVDF membrane with 220 nm pore size at 5000× magnification, (**c**) PVDF membrane with 220 nm pore size sandwiched between a monolayer of electrospun PVDF fibers at 1000× magnification, (**d**) PVDF membrane with 220 nm pore size, sandwiched between a bilayer of electrospun PVDF fibers at 500× magnification, (**e**) PVDF membrane with 450 nm pore size at 2000× magnification, (**f**) PVDF membrane with 450 nm pore size, sandwiched between a monolayer of electrospun fibers, at 500× magnification, and (**g**) PVDF membrane with 450 nm pore size, sandwiched between a bilayer of electrospun fibers, at 500× magnification.

The surface morphology of the PVDF fiber-membranes was observed by SEM and compared with that of the PP separators, as shown in Figure 5. The PP separators have an average pore size of ~570 nm. The pore distribution of the PVDF membranes only (i.e., without fibers) is dense. After sandwiching the PVDF membranes between the fabricated fibers, their average pore size and pore distribution were reduced. Using Equation (1), the porosity of the separators were calculated based on the density of PP (0.946 g/cm^3^) and the PVDF membrane (1.78 g/cm^3^). The porosity of the PP separator was found to be ~62%, that of PVDF membrane with a 220 nm pore size was ~73%, and that of PVDF membrane with 220 nm pore size was ~71%. After sandwiching the 220 nm PVDF membrane between the monolayer and bilayer of electrospun fibers, the porosity was relatively maintained at ~71% and ~70%, respectively. Meanwhile, when the 450 nm PVDF membrane was sandwiched between the monolayer and bilayer of electrospun fibers, the porosity became ~70% and ~63%, respectively. Therefore, the porosity of the PVDF fiber-membranes was relatively higher and more stable than that of the PP separators. This is attributable to the pore density of the PVDF fiber-membrane, which was superior to that of the PP separator.

### 3.2. Surface Analysis of A4 Paper, Rice Paper, Nonwoven Fabric, and Carbon Synthetic Fabric Coated with Sol-Gel

**Figure 6:**
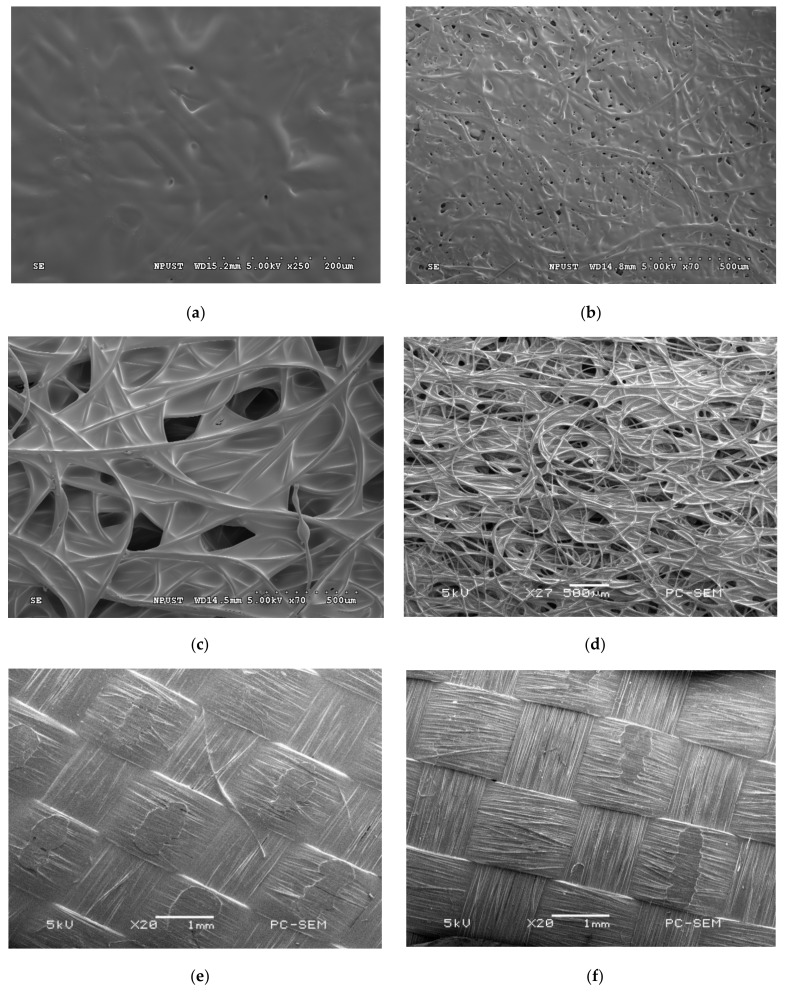
SEM images of nano-ceramic solutions coated on the four substrates; (**a**) BX900 coated on A4 paper, (**b**) BX900 coated on rice paper, at 70× magnification, (**c**) BX100 coated on the nonwoven fabric, (**d**) BX900 coated on nonwoven fabric, at 27× magnification, (**e**) BX100 coated on the carbon synthetic fabric at 20× magnification, and (**f**) BX900 coated on the carbon synthetic fabric at 20× magnification.

The surface morphology of the four substrates (A4 paper, rice paper, nonwoven fabric, and carbon synthetic fiber, was observed by SEM, and their porosities were calculated using Equation (1). Figure 6 shows the SEM images of the substrates with sol-gel coating. When BX900 was coated on A4 paper, the porosity was determined to be ~32%. The surface had almost no pores. The porosity of the rice paper after coating with BX900 was ~37%. The porosity of nonwoven fabric was ~63% and ~64% after coating with BX100 and BX900, respectively. Because of its large pore size and excellent pore distribution, the nonwoven fabric maintained excellent porosity after coating. After the carbon synthetic fabric was coated with BX100 and BX900, the porosity was ~56% and ~59%, respectively. The results showed that the pores of the substrates were reduced after the sol-gel coating.

### 3.3. Thermal Stability Analysis of PP Separator and PVDF Fiber-Membranes

**Figure 7:**
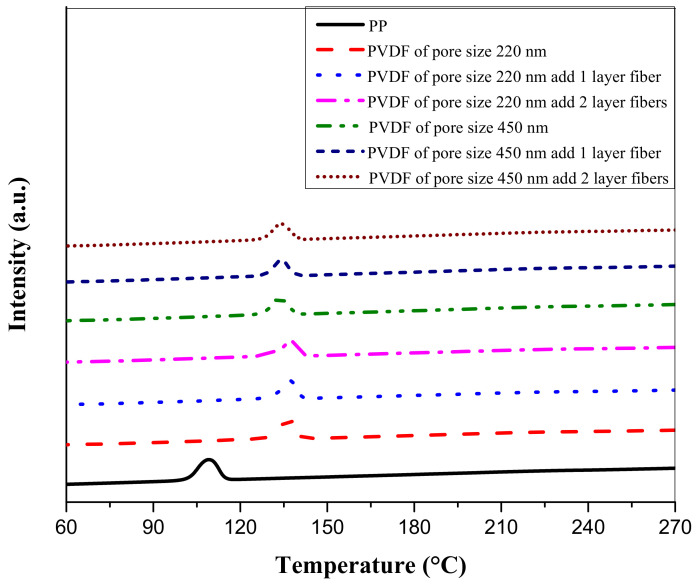
Comparison of exothermic peaks of PP separator and PVDF fiber-membranes.

The DSC measurements are shown in Table 4. The PP separator began to melt at ~150 °C and had an endothermic peak at ~165 °C. The PVDF fiber-membranes melted at ~165 °C and had an endothermic peak at ~170–173 °C. They melted completely at ~180 °C because the high electronegativity of the fluorine atom and the high bond dissociation of the C–F bond chain provided thermal stability to the fluoropolymer. Thus, the thermal stability of the fluoropolymer was superior to that of the hydrocarbon polymer [18]. The PVDF membrane was pyrolyzed due to the phenomenon of dehydrofluorination, which led to the formation of the C–C double bonds or cross-linking of polymers [19]. Next, according to the DSC measurements shown in Figure 7, the PP separator had an exothermic peak at ~110 °C while the PVDF fiber-membranes had exothermic peaks from ~130 to 140 °C. The appearance of an exothermic peak indicates the occurrence of crystallization reaction. In this experiment, DSC was used to measure and determine the highest melting point of a material to ensure good thermal stability under normal use. For future study, thermal gravimetric analysis (TGA) can be used to measure and characterize more properties of the separators.

Crystallinity was calculated by Equation (2) using the $∆H_{f}^{0}$ values of 104.7 J/g for PVDF and 207 J/g for PP [20,21]. According to Table 4, the crystallinity of the PP separator was ~16% while that of the PVDF fiber-membranes ranged from ~18% to ~23%.

**Figure 8:**
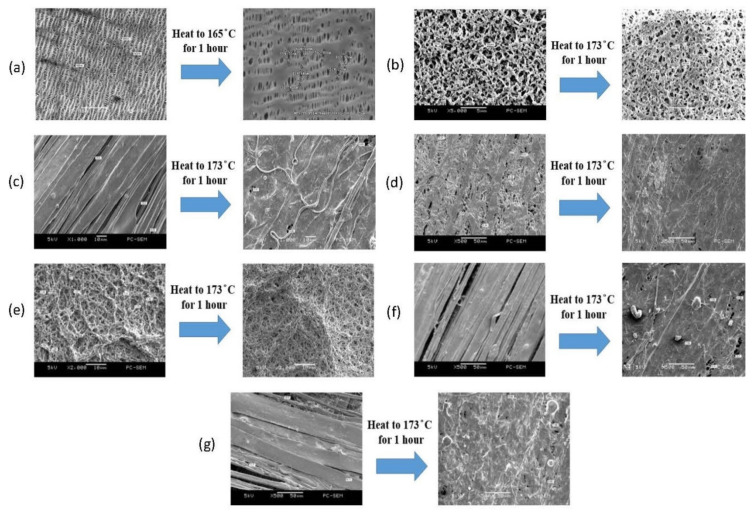
SEM images showing the effect of heating on the pore size of the PP separator and PVDF fiber-membranes; (**a**) pore size of PP separator when heated to 165 °C for 1 h, at 5000× magnification, (**b**) pore size of the 220 nm PVDF membrane when heated to 173 °C for 1 h, at 5000× magnification, (**c**) pore size of the 220 nm PVDF membrane sandwiched between a monolayer of electrospun fibers when heated to 173 °C for 1 h, at 1000× magnification, (**d**) pore size of the 220 nm PVDF membrane sandwiched between a bilayer of electrospun fibers when heated to 173 °C for 1 h, at 500× magnification, (**e**) pore size of the 450 nm PVDF membrane heated to 173 °C for 1 h, at 2000× magnification, (**f**) pore size of the 450 nm PVDF membrane sandwiched between a monolayer of electrospun fibers when heated to 173 °C for 1 h, at 500× magnification, and (**g**) pore size of the 450 nm PVDF membrane sandwiched by a bilayer of electrospun fibers when heated to 173 °C for 1 h, at 500× magnification.

Based on Figure 8, the pores of the PP separator were reduced when the PP separator was heated to 165 °C, as the pores tend to close at high temperatures. Because the PP separator has thermally closed pores, electrode contact was prevented. When the 220 nm and 450 nm PVDF membranes were sandwiched between layers of electrospun fibers and heated to 173 °C, the fibers melted, thus filling the pores of the PVDF membranes and resulting in thermally closed pores.

### 3.4. Thermal Stability Analysis of A4 Paper, Rice Paper, Nonwoven Fabric, and Carbon Synthetic Fabric Coated with Sol-Gel

Sol-gel was coated on A4 paper, rice paper, nonwoven fabric, and carbon synthetic fabric. The DSC result revealed no melting peak within 270 °C, thus indicating that the materials did not shrink or melt at high temperatures, which suggests thermal stability. This result. can be attributed to the sol-gel coating, which contained the nanoceramic powders Al_2_O_3_ and SiO_2_, suggesting that the ceramic coating can effectively improve the thermal stability of the separators [22,23].

### 3.5. Ion Conductivity Analysis of PP Separator and PVDF Fiber-Membranes

For the AC resistance test, the separator and stainless-steel caps were assembled into a button battery. The high frequency swept to low frequency, and the current signal was converted into electrochemical impedance spectra (EIS) using software. The EIS result are listed in Table 5. The ion conductivities of the PP separator and PVDF fiber membranes, calculated using Equation (3), are shown in Table 5. The results showed that the PP separator has an ion conductivity of ~0.29 mS/cm while that of the PVDF fiber-membranes ranged from 0.44 to 0.66 mS/cm. Clearly, the ion conductivity of the PVDF fiber-membranes was relatively superior to that of the PP separator. This can be attributed to the fact that PP is a nonpolar polymer material with a poor affinity for electrolytes, while the PVDF membrane is a highly polar material with high porosity [24].

### 3.6. Ion Conductivity Analysis of A4 Paper, Rice Paper, Nonwoven Fabric, and Carbon Synthetic Fabric Coated with Sol-Gel

The ion conductivities of A4 paper, rice paper, nonwoven fabric, and carbon synthetic fabric coated with sol-gel were calculated using Equation (3). For AC, the resistance test, the separator and stainless-steel caps were assembled into a button battery. The high frequency swept to low frequency, and the current signal was converted into EIS using software. EIS spectra were analyzed by using the formula as shown in Equation (3).

The EIS data showing the ion conductivities of the sol-gel coated A4 paper, rice paper, nonwoven fabric, and carbon synthetic fabric are shown in Table 6.

Based on Table 6, the rice paper obtained an electric impedance comparable to that of the PP separator and an ion conductivity of ~0.48 mS/cm. Because rice paper is thin, it exhibited better ion conduction efficiency. The electrical impedance of A4 paper and nonwoven fabric were between ~21 and 26 Ω. The ion conductivity of A4 paper was ~0.15 mS/cm while that of the nonwoven fabric was ~0.39 mS/cm. Although the A4 paper was thinner than the nonwoven fabric, its pore structure was less, so the nonwoven’s ion conductivity was higher because of its large pores. The ion conductivity of carbon synthetic fabric was ~0.22 mS/cm while its electrical impedance was 58 Ω. These values are due to the relative thickness of the carbon synthetic fabric.

A4 paper coated with sol-gel exhibited the highest electrical impendence because of its small pore structure. The pores were almost filled after the addition of the sol-gel coating. Upon coating, the ion conductivity of the rice paper and nonwoven fabric were ~0.16 mS/cm and ~0.96 mS/cm, respectively. Because the ratio of BX900 was 9:1, the sol-gel had a greater amount than the organic polymer resin, resulting in low adhesion. The nonwoven fabric had an excellent distribution of structurally large pores, so many pores were retained after sol-gel coating. The ion conductivity of the carbon synthetic fabric was ~0.47 mS/cm after coating with sol-gel. Thus, after coating with BX900, the ion conductivity of the nonwoven and carbon synthetic fabrics were relatively superior to that of the PP separator. Finally, based on the discussion above and results in Table 6, the porosity and structure of the pores significantly affected ion transport efficiency.

### 3.7. Redox Reactions of PP Separator and PVDF Fiber-Membranes

**Figure 9:**
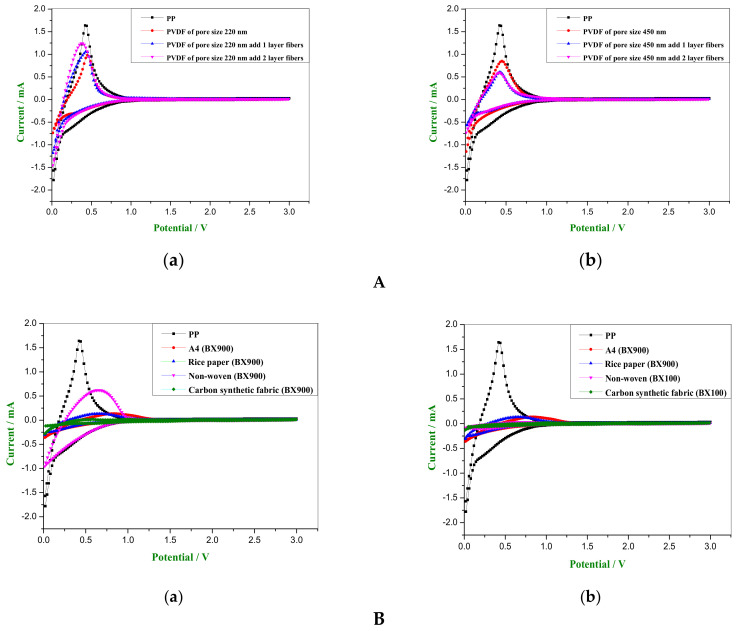
(**A**). Comparison of CV graphs of the separators: (**a**) PP separator versus 220 nm PVDF fiber-membranes sandwiched between mono- and bi-layers of electrospun fibers; (**b**) PP separator versus 450 nm PVDF fiber- membranes sandwiched between mono- and bi-layers of electrospun fibers; (**B**). Comparison of CV graphs: (**a**) CV graphs of substrate with best conductivities after coating with sol-gel; (**b**) CV graphs of the substrate after sol-gel coating showing that the ion conductivity is similar to the PP separator.

The electrochemical stability of separators was measured by cyclic voltammetry (CV). The CV scanned the current signal in the 0.01 V~3 V range and selected the third loop to plot. The CV graphs are shown in Figure 9A. It can be seen that when the voltage was swept from 3 V to 0 V it represented the reduction reaction of lithium ions. At this time, the PP separator had a peak at ~0.021 V. The 220 nm PVDF fiber-membrane had a peak at ~0.012 V. The 450 nm PVDF fiber-membrane had a peak between 0.01 V and 0.03 V. When the voltage was swept from 0 V to 3 V, it represented the oxidation reaction of lithium ions. At this time, the PP separator had a peak at ~0.042 V. The 220 nm PVDF fiber-membrane had a peak between 0.38 V to 0.44 V. The 450 nm PVDF fiber-membrane had a peak between 0.42 V to 0.44 V. Therefore, the oxidation peak of the 220 nm PVDF fiber-membrane was higher than the 450 nm PVDF fiber-membrane. The oxidation reaction characteristics of the 220 nm PVDF fiber-membrane were second to those of the PP separator. In Figure 9(Aa), it can be observed that the current of the 220 nm PVDF fiber-membrane increased sharply at ~0.6 V. The current of the PP separator increased sharply at ~0.7 V. Thus, the 220 nm PVDF fiber-membrane showed a better anode stability than the PP separator because it had a better interfacial compatibility with the electrolyte [9].

### 3.8. Redox Reactions of A4 Paper, Rice Paper, Nonwoven Fabric and Carbon Synthetic Fabric Coated with Sol-Gel

The CV scanned the current signal in the 0.01 V~3 V range and selected the third loop to plot. The CV graphs are shown in Figure 9B. It can be seen that when the voltage was swept from 3 V to 0 V, it represented the reduction reaction of lithium ions. At this time, the peak of the nonwoven fabric with BX900 coating appeared at ~0.034 V. When the voltage was swept from 0 V to 3 V, it represented the oxidation reaction of lithium ions. The peak appeared at ~0.63 V. The oxidation peak of the nonwoven fabric with BX900 coating was most remarkable, as shown in Figure 9(Ba). Although the ion conductivity of the nonwoven and the carbon synthetic fabrics after coating with the BX100 were similar to the PP separator, there was no significant redox reaction. The CV graph is shown in Figure 9(Bb). Because the BX100 ratio was 1:1, it had stronger adhesion. The pores of the nonwoven fabric and the carbon fiber cloth were filled more. At the third cycle, the ions could not be embedded in the electrode easily, so the charge could not be stored efficiently.

### 3.9. Cycle Performance of PP Separator and PVDF Fiber-Membranes

**Figure 10:**
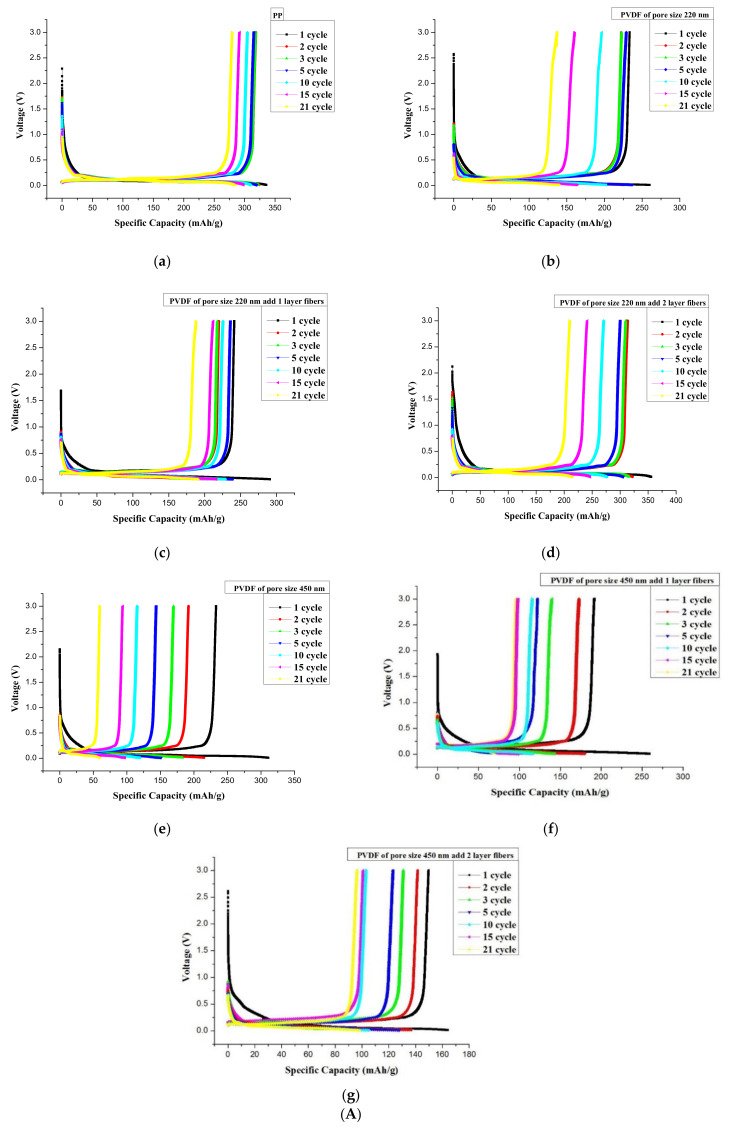

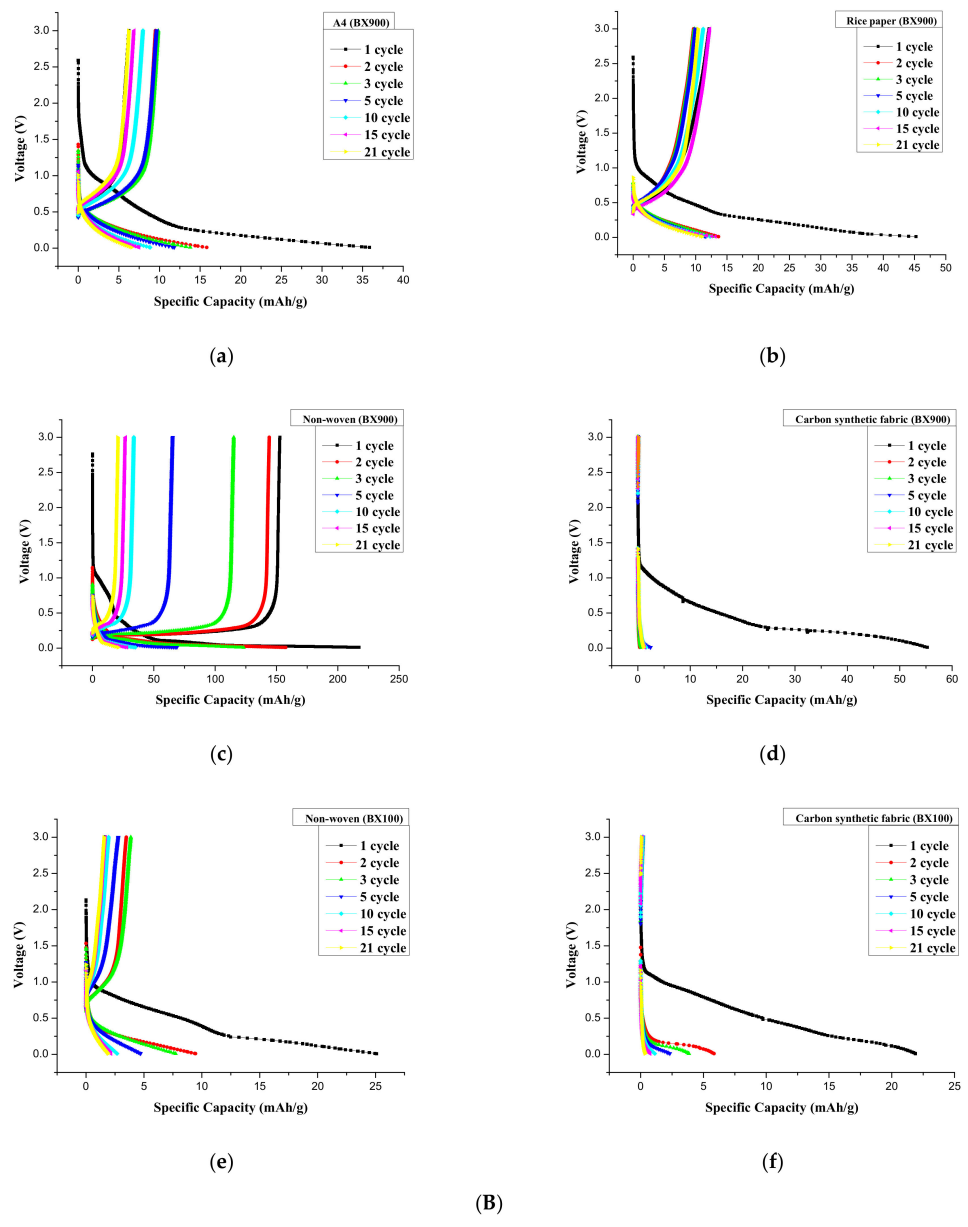
(**A**) Comparison of charge-discharge curves; (**a**) PP separator, (**b**) 220 nm PVDF membrane, (**c**) 220 nm PVDF membrane sandwiched in fiber monolayer, (**d**) 220 nm PVDF membrane sandwiched in fiber bilayer, (**e**) 450 nm PVDF membrane, (**f**) 450 nm PVDF membrane sandwiched in fiber monolayer, and (**g**) 450 nm PVDF membrane sandwiched in fiber bilayer, (**B**) Comparison of charge and discharge curves; (**a**) A4 paper with BX900 coating, (**b**) rice paper with BX900 coating, (**c**) nonwoven fabric with BX900 coating, (**d**) carbon synthetic fabric with BX900 coating, (**e**) nonwoven fabric with BX100 coating, and (**f**) carbon synthetic fabric with BX100 coating.

The different separators—PP separator and 220 nm and 450 nm PVDF fiber-membranes—were assembled to form a button battery to determine their cycle performance. The charge and discharge test of the batteries was carried out for 21 cycles at the 0.2 C current density. We selected 1, 2, 3, 5, 10, 15, and 21 cycles to construct the graphs, as shown in Figure 10A.

The lithium-ion battery using the PP separator registered a capacity of ~331 mAh/g. The lithium-ion battery with the 220 nm PVDF fiber-membrane had a capacity between 270 and 356 mAh/g. When the electrospun fibers were used to sandwich the PDF membrane in a bilayer, the battery capacity tended to increase. The lithium-ion battery that used the 450 nm PVDF fiber-membrane registered a capacity between 168 and 313 mAh/g. The capacitance decreased when it was sandwiched between the electrospun fiber layers.

When the 220 nm PVDF membrane was sandwiched between a bilayer of electrospun fibers, the capacity turned out better than the PP separator. This is because the dipole moment was regularly arranged along the polarization direction inside the fiber to generate more charge. The charge is embedded in the electrode through the electrolyte, thus effectively increasing the battery capacity.

For the cycle performance determination, the 220 nm PVDF membrane sandwiched in a bilayer of electrospun fibers was used to assemble a button battery. The charge and discharge test were performed at a current density of 0.2 C for 100 cycles. The first discharge capacity of the PVDF fiber-membrane (in a bilayer fiber sandwich) was better than that of the PP separator with a first-cycle discharge capacity of ~356 mAh/g. The first cycle discharge capacity was superior to that of the PP separator. The porosity of the PP separator was ~62%. The PVDF membrane porosity was between 63 and 70%. Therefore, the porosity of the PVDF fiber membrane was better than that of PP separator. The PP separator began to melt at ~150 °C and had an endothermic peak at ~165 °C. The PVDF fiber membranes melted at ~165 °C, had an endothermic peak at ~173 °C, and melted completely at ~180 °C. In addition, the analysis results showed that the ion conductivity of the PP separator was ~0.29 mS/cm. The ion conductivity of PVDF membranes was between 0.44 and 0.66 mS/cm. The ion conductivity of PVDF fiber membranes was superior to that of the PP separator. The PP was used as separator membrane for a lithium-ion battery that had a capacity of ~331 mAh/g. The PVDF was used as separator membrane for a lithium-ion battery having a capacity between 270 and 356 mAh/g. The first-cycle discharge capacity was ~356 mAh/g, which was better than that of the PP. To sum up, PVDF as separator membrane showed a better potential application for a lithium battery than did a commercial PP.

### 3.10. Cycle Performance of A4 Paper, Rice Paper, Nonwoven Fabric, and Carbon Synthetic Fabric Coated with Sol-Gel

A4 paper, rice paper, nonwoven fabric and carbon synthetic fabric were coated with sol-gel and used as separators in an assembled button battery. The charge and discharge test of the assembled battery was carried out for 21 cycles at the 0.2 C current density. The 1, 2, 3, 5, 10, 15, and 21 cycles were selected to construct the graphs shown in Figure 10B. The lithium ion battery using A4 paper coated with BX900 registered a capacity of ~36.3 mAh/g. The lithium-ion battery using rice paper coated with BX900 registered a capacity of ~46 mAh/g. Finally, the lithium-ion battery using nonwoven fabric with BX900 and BX100 coating had a capacity of ~224 mAh/g and ~25 mAh/g, respectively.

## 4. Discussion

Innovations in battery-separator technology could come in the form of the materials used or the processes used to fabricate the materials or both materials and processes. In this study, when the PVDF membranes with pore diameters of 220 nm and 450 nm were observed by a SEM, the pore structure distribution appeared to be dense. After sandwiching the PVDF membranes with electrospun fibers, the porosity was maintained between 63% to 71%. The ion conductivity was between 0.45 and 0.66 mS/cm. The data show that PVDF was a highly polar material with good affinity for the electrolyte. The structure of the fiber-membrane has a high porosity which enhanced the ion transport efficiency.

The melting temperature of the PVDF fiber-membrane is ~165 °C, which was higher than the melting temperature of PP (at ~150 °C). Thus, the thermal stability of the fluoropolymers was better than that of the hydrocarbon polymers. The high electronegativity of the fluorine atom and the high bond dissociation of the C–F bond provided thermal stability to the fluoropolymer.

When the PVDF membrane with a pore size of 220 nm was sandwiched between a bilayer of electrospun fibers, the anode acquired good stability. The first cycle discharge capacity was ~356 mAh/g which was better than that of the PP. This suggests that the dipole moment is regularly arranged inside the fiber after polarization by the electric field. The transmission of the charge could increase the specific capacitance effectively through the electrolyte.

A4 paper, rice paper, nonwoven fabric, and carbon synthetic fabric coated with sol-gel resulted in no melting peaks within 270 °C through DSC analysis, suggesting that the sol-gel containing Al_2_O_3_ and SiO_2_ could enhance the thermal stability of the materials when used as separators.

The BX900 coating on nonwoven fabric with a porosity of ~64% and ion conductivity of ~0.96 mS/cm emerged as the best. The oxidation peak is the most significant. The specific capacitance reached ~224 mAh/g, but its cycle performance was poor. The capacity dropped to ~25 mAh/g after 21 cycles. The carbon synthetic fabric coated with sol-gel and used as a lithium-ion battery separator showed multiple charge and discharge measurements. The charge and discharge curves are shown in Figure 10. Because the carbon synthetic fabric was thick to begin with, sol-gel coating further increased its thickness. Thus, the ions could not be embedded and removed successfully, so the electrodes could not be charged and discharged.

## 5. Conclusions

The search for a better and safer battery technology for electric vehicles is an active area of research, innovation and development. The overarching concern for environmental protection, energy sustainability and cost will continue to fuel these efforts. Better separators could substantially improve the performance of lithium-ion batteries, lengthen their cycle life, and promote their safe use. According to the findings of the investigation, the 220 nm PVDF membrane sandwiched between a bilayer of electrospun fibers showed excellent ionic conductivity, porosity and endothermic peak along with better specific capacitance, higher melting temperature and a stable cycle performance. The sol-gel coated nonwoven fabric had notable thermal stability despite a lower specific capacitance and no peak below 270 °C. The present study represents a significant step in the innovation of the materials and processes in the fabrication of LIB separators. These innovations could accelerate the development of electric vehicles as the preferred mode of transportation, leading to a much cleaner and healthier environment for everyone.

## Figures and Tables

**Table 1:** Details of chemicals used in the preparation of PVDF solution.

Chemical	Formula	Molecular Weight (g/mol)
PVDF	(C_12_H_13_NO_3_)_n_	534,000
DMSO	C_2_H_6_OS	78.13
Acetone	C_3_H_6_O	58.08
Surfactant	C_12_H_25_NaSO_4_	288.378

**Table 2:** PVDF solution configuration.

Solution A		Solution B	
PVDF powder (g)	Acetone (g)	DMSO (g)	Surfactant (g)
0.90	2.50	2.50	0.20

**Table 3:** NFES parameters for the fabrication of PVDF fibers.

Equipment Part/Aspect	Parameter
Needle size	Length, 12.7 mm; inside diameter, 0.25 mm
Collecting distance	1 mm
Tangential speed	2618.10 mm/s
Pump feed rate	0.15 mL/h
Voltage field	1.40 × 10^7^ V/m

**Table 4:** Comparison of the Crystallinity of PP Separator and PVDF Fiber-membranes.

Separator	T_m_ (°C)	$∆H_{f}\;(J/g)$	Crystallinity (%)
PP	164.8	33.33	16.1
PVDF (220 nm) membrane only	171.1	23.84	22.77
PVDF (220 nm) with a monolayer of fibers	170.7	22.18	21.18
PVDF (220 nm) with a bilayer of fibers	170.8	21.23	20.28
PVDF (450 nm) membrane only	171.0	21.16	20.21
PVDF (450 nm) with a monolayer of fibers	170.3	19.32	18.45
PVDF (450 nm) with a bilayer of fibers	171.1	23.1	22.06

**Table 5:** Comparison of ion conductivity of PP separator and PVDF fiber membranes.

Separator	Thickness (μm)	Resistance (Ω)	Ionic Conductivity mS/cm (Error)
PP	28	3.79	0.29 (−0.01–0.01)
PVDF (220 nm) membrane only	128	7.62	0.66 (−0.02–0.02)
PVDF (220 nm) with monolayer of fibers	144	12.88	0.44 (−0.04–0.02)
PVDF (220 nm) with bilayer of fibers	161	11.06	0.57 (−0.01–0.01)
PVDF (450 nm) membrane only	120	7.24	0.65 (−0.03–0.01)
PVDF (450 nm) with monolayer of fibers	142	11.98	0.46 (−0.01–0.01)
PVDF (450 nm) with bilayer of fibers	164	12.64	0.51 (−0.01–0.02)

**Table 6:** The ion conductivity of A4 paper, rice paper, nonwoven fabric, and carbon synthetic fabric.

Sample	Thickness (μm)	Resistance (Ω)	Ionic Conductivity (mS/cm)
A4 paper only	105	26.78	0.15
A4 (with BX100)	142	208.75	0.026
A4 (with BX300)	120	139.49	0.034
A4 (with BX900)	117	92.99	0.049
Rice paper only	86	7.10	0.48
Rice paper (with BX100)	102	32.39	0.12
Rice paper (with BX300)	105	59.33	0.072
Rice paper (with BX900)	89	22.13	0.16
Nonwoven fabric only	211	21.42	0.39
Nonwoven fabric (with BX100)	365	47.70	0.30
Nonwoven fabric (with BX300)	349	26.20	0.52
Nonwoven fabric (with BX900)	347	14.30	0.96
Carbon Synthetic fabric only	327	57.93	0.22
Carbon synthetic fabric (with BX100)	452	48.36	0.36
Carbon synthetic fabric (with BX300)	445	47.07	0.37
Carbon synthetic fabric (with BX900)	430	36.26	0.47

## Data Availability

Not applicable.
